# Genetic diversity of toll-like receptor genes in the vulnerable Chinese egret (*Egretta eulophotes*)

**DOI:** 10.1371/journal.pone.0233714

**Published:** 2020-05-29

**Authors:** Wei Xu, Xiaoping Zhou, Wenzhen Fang, Xiaolin Chen

**Affiliations:** Key Laboratory of the Ministry of Education for Coastal and Wetland Ecosystems, College of the Environment and Ecology, Xiamen University, Xiamen, Fujian, People’s Republic of China; University of Regina, CANADA

## Abstract

Toll-like receptor (TLR) genes have recently been employed to assess genetic diversity, as they can be used to infer both demographic history and adaptation to environments with different pathogen pressure. Here, we sampled 120 individuals of the Chinese egret (*Egretta eulophotes*), a globally vulnerable species, from four breeding populations across China. We assessed the levels of genetic diversity, selection pressure, and population differentiation at seven TLR loci (TLR1LB, TLR2A, TLR3, TLR4, TLR5, TLR7, and TLR15). Using a variety of metrics (SNPs, heterozygosity, nucleotides, haplotypes), our analyses showed that genetic diversity was lower at 4 of the 7 TLR loci in the vulnerable Chinese egret compared to the more common little egret (*Egretta garzetta*). The selection test indicated TLRs, except for TLR5, were under purifying selection in TLR evolution, suggesting that low TLR genetic diversity in the Chinese egret may be caused by purifying selection. Moreover, analysis of molecular variance indicated low but significant population differentiation among four populations at all of the TLR loci in this egret. However, some comparisons based on fixation index analyses did not show significant population differentiation, and Bayesian clustering showed admixture. Our finding suggested that these four populations of the Chinese egret in China may be considered a single unit for conservation planning. These results, the new report of TLR genetic diversity in a long-distance migratory vulnerable Ardeid species, will provide fundamental TLR information for further studies on the conservation genetics of the Chinese egret and other Ardeids.

## Introduction

Loss of genetic variability and inbreeding depression may consequentially increase extinction risk for threatened species by decreasing reproductive fitness and adaptive potential and increasing disease susceptibility in a changing environment [1]. Thus, understanding genetic diversity and the spatial structure of threatened populations is crucial for developing effective conservation and management plans [2, 3].

Traditionally, neutral molecular markers (such as microsatellites and mtDNA) have been effectively used to assess genetic variation, with further analysis to provide demographic history and determine population structure [4–6]. However, neutral loci may not be relevant in studying processes affecting functional diversity [7], and such markers cannot reflect how populations adapt to different environments. Increasingly many studies have focused on adaptive genes such as the major histocompatibility complex (MHC) and toll-like receptors (TLRs) that are affected by both demographic and selective factors [8–10]. MHC genes provide information concerning individual and population viability due to their direct association with immune function [11, 12]. However, analyses of MHC loci may not be very successful in many non-model species, as their high numbers of pseudogenes and duplications will interfere with genetic diversity estimates. This has affected estimates in passerine birds [12, 13]. In addition, previous studies on humans (and potentially other vertebrates) have found that at least half of the genetic basis of inter individual variability in immune responses to pathogens is considered to be a consequence of non-MHC genes [14].

TLRs are an ancient family of innate-immunity genes that recognize and bind to a variety of Pathogen-Associated Molecular Patterns (PAMPs). The structure of TLRs consists of three components: the characteristic horseshoe-shaped ectodomain that directly contacts pathogens and leads to most variations, a transmembrane domain, and an intracellular Toll-interleukin 1 receptor (TIR) domain that enables downstream signal transmission [15, 16]. As an essential part of the first line of defense against pathogens, TLRs initiate the innate and adaptive aspects of the immune response through intracellular signaling [17]. Compared to MHC, TLRs are relatively easy to obtain and can be more advantageous in assaying immune-gene heterozygosity by allowing diversity estimates for multiple genes. Based on these advantages, TLRs have been increasingly used to estimate adaptive genetic diversity in bird species [7, 12, 13, 18–23].

To date, 10 avian TLR family members have been identified based on their functions and sequences in the Red jungle fowl (*Gallus gallus*, NCBI: NC_006091.5) and other avian species. According to Wang’s classification [24], the 10 avian TLR family members can be divided into two categories: the single-domain TLRs possessing a complete asparagine ladder (TLR3, TLR5, TLR7, TLR15, and TLR21) and the three-domain TLRs with the ladder interrupted in the central part of the ectodomain (TLR1LA, TLR1LB, TLR2A, TLR2B, and TLR4). Various kinds of TLR family members recognize different kinds of pathogens; TLR1 can form a heterodimer with TLR2 to detect lipopeptides; TLR3 detects dsRNA; TLR7 binds ssRNA; TLR4 and TLR5 detect lipopolysaccharides and bacterial flagellin, respectively. In the TLR family, TLR 15 has only been identified in avian and reptilian species, and its feature activation mechanism can support a novel function in recognition of extracellular proteases. TLR21 is homologous to mammalian TLR9, which recognizes unmethylated CpG DNA [15].

The Chinese egret (*Egretta eulophotes*) is a species of migratory colonial wading bird in the family of Ardeidae that overwinters in the Philippines, Vietnam, Malaysia, Singapore, Indonesia, and Brunei while breeding in Russia, North Korea, South Korea, and China [25, 26]. Currently this egret is listed as a vulnerable species, with an estimated global population of 2,500 to 9,999 mature individuals [25]. Today, the main threat to this species is the ongoing loss and degradation of coastal wetland habitats and uninhabited offshore breeding islands due to human activities, such as reclamation, infrastructure, pollution, industry, aquaculture, agriculture, excessive fishing, alien species, climate change, egg collection for food, tourism disturbances and illegal hunting [26, 27]. In our previous studies, we found that the genetic diversity of the Chinese egret was relatively high in mtDNA and at a low level in MHC class II DAB genes [3, 5], and their populations had low but significant amounts of genetic differentiation with weak geographical structure. To expand our previous work, this study aimed specifically to: 1) access TLR genetic diversity in the vulnerable Chinese egret and compare the results with a common Ardeid species, the little egret (*Egretta garzetta*), and other reported avian species; 2) analyze selection in the evolution of TLRs in this egret; and 3) detect population differentiation and population structure for TLRs in the Chinese egret and delineate a conservation unit for this vulnerable species across China. We hypothesize that both SNPs and genetic diversity as indicated by heterozygosity, nucleotides, and haplotypes of toll-like receptor genes in the vulnerable species, the Chinese egret, are lower than those in the more common species, the little egret, due to the purifying/negative selection acting on the TLRs. To our knowledge, this is the new study of TLR genetic diversity in a long-distance migratory vulnerable Ardeid species.

## Materials and methods

### Ethics statement

This research and all procedures involving collection of animal tissue in the wild were approved by the Administration Center for Wildlife Conservation in Fujian Province (FJWCA-1208). The scientific license for access to the study site was issued by the Administration Department of Xiamen Egret Natural Reserve (XMENR-1005). Feathers were plucked and blood samples (~0.5mL) were collected from nestlings (aged around 10 days) of the Chinese egret (*Egretta eulophotes*) or little egret (*E*. *garzetta*). Blood samples were obtained by puncturing the wing vein and using a syringe to withdraw. The nestlings were immediately returned to the nest after stanching the wound with cotton. Collection of samples (feathers or blood samples of nestlings) was conducted in the morning during the breeding season, and visits to a breeding colony were restricted to a maximum of two hours per day.

### Study areas and sample collection

**Fig 1:**
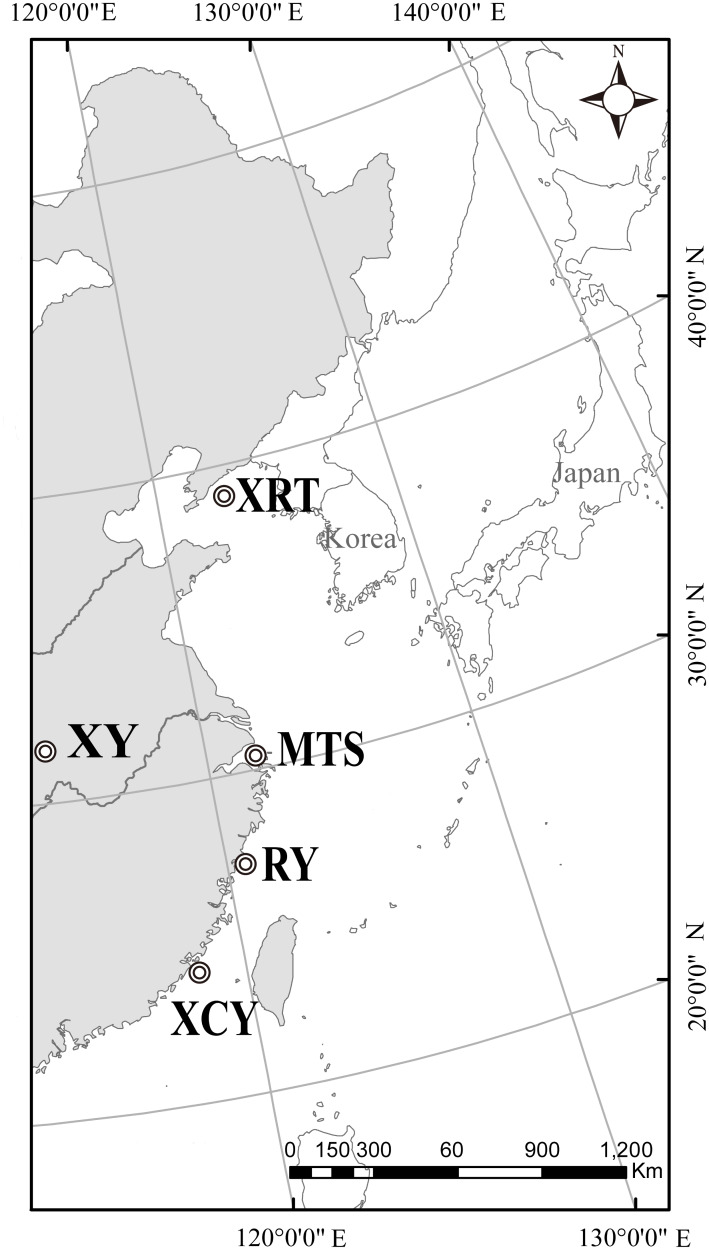
Geographical locations of the sampled Chinese egret populations and little egret populations in China. *XRT* Xingrentuo, *XY* Xinyang, *MTS* Mantoushan, *RY* Riyu, *XCY* Xiaocaiyu.

Samples of the Chinese egret were collected from four archipelago populations: Xingrentuo (XRT; 39°31'N, 123°03'E), Mantoushan (MTS; 30°13'N, 121°53'E), Riyu (RY; 27°01'N, 120°25'E) and Xiaocaiyu (XCY; 23°48'N, 117°45'E). Samples of little egret were obtained from two sites in China, Xinyang (XY; 32°07'N, 114°04'E) and Mantoushan (MTS; 30°13'N, 121°53'E). These offshore islands (XRT, MTS, RY and XCY) represent most of the known breeding sites of the Chinese egret across China (Fig 1). To avoid the possibility of sampling siblings, feathers or blood samples were randomly obtained from one of the nestlings in each nest. Finally, we randomly selected 20 individuals of the little egret (10 individuals per location) and 120 individuals of the Chinese egret (30 individuals per location). All of the feather samples were kept in 75% ethanol and stored at -80°C, while the blood samples were kept in 1% EDTA-Na2 at -80°C until extraction.

### Genomic DNA isolation, amplification, and sequencing

DNA was extracted using the universal Genomic DNA Extraction Kit Ver. 3.0 (TaKaRa) from samples (feathers or blood samples of nestlings). The Leucine Rich Repeats (LRRs) domains of TLR genes were targeted from the genome of the Chinese egret (our unpublished data), and the primer set for amplifying part of the LRRs region (TLR1LB, TLR2A, TLR3, TLR4, TLR5, TLR7, TLR15) was designed using the online resource Primer-BLAST (S1 Table). Amplifications were performed in a total volume of 25 μl containing 1 μl (approximately 100 ng) extracted genomic DNA, 2 × EasyTaq PCR SuperMix (TransGen Biotech, Beijing, China) and 1 μl 10 μmol/L forward and reverse primers. We set the PCR thermocycler program as follows: 94°C for 5 min as initial denaturalization, followed by 35 cycles of 94°C for 30 s, 60°C for 30s, 72°C for 90s, and a final extension at 72°C for 10 min. All of the PCR products were purified using an Agarose Gel DNA Purification Kit (GENERAY) and sequenced in both directions on an automatic sequencer (ABI PRISM 3730) by Boray Biotechnology Co., Ltd (Xiamen, China). All of the heterozygous individuals for any TLR genes were re-amplified and cloned to confirm SNP sites (cloned using the *Peasy*-T1 Cloning kit (TransGen Biotech, Beijing, China), and we obtained the most accurate haplotypes of different TLR genes in this research compared with other studies using software inference. In this study, any individual included in the analysis (and reported in the results) had no missing SNP data.

### Data analysis

After we aligned all of the sequences by using MEGA version 7.0 [28], phylogenetic analysis was performed using the neighbor-joining method, with 5000 bootstrap replicates used to verify the classification (S1 Fig). The MEGA was also used to determine synonymous and non-synonymous SNP variations by translating the TLR gene nucleotide sequences to the amino acid sequences. GenALEx 6.503 [29] was used to calculate observed heterozygosity (Ho), mean expected heterozygosity (He), unbiased expected heterozygosity (uHe) and Hardy-Weinberg Equilibrium (HWE). The number of SNPs, the number of haplotypes (*h*), the nucleotide diversity (π) among sequences, Watterson’s estimator of the population mutation rate (θw) and the average number of nucleotide differences between alleles (k) were estimated by using DNAsp V6.11.01 [30]. Deviation from expectations of neutral evolution was also estimated through the Tajima’s D test by using DNAsp V6.11.01. For comparison with other avian data from published studies, we used two genetic indices, *h* and π, which were plotted graphically in Microsoft Excel 2016.

DataMonkey (http://www.datamonkey.org) was used to search for positively selected sites by two methods: SLAC, a fast and conservative method [31] and the FUBAR algorithm, which is more robust and much faster than other available selection tests based on random effect likelihood (REL) methods [15, 32]. The sites were interpreted as having positive selection only when the results were supported by both independent methods. To minimize the overestimation of positively selected codons, codons with *p* values < 0.1 for SLAC and with posterior probabilities > 0.9 for FUBAR were considered as candidates to be under positive selection. In addition, the mixed-effects model of evolution (MEME) was used to test for episodic diversifying selection in the TLRs by using DataMonkey (default α = 0.1 as significance threshold). MEME is an extension of FEL and most appropriate to detect episodic diversifying selection affecting individual codon sites. Pairwise comparison F_ST_ (9999 permutations), analysis of molecular variance (AMOVA) (9999 permutations) and Mantel tests were carried out with GenALEx 6.503. The Mantel test was performed based on 9999 permutations to investigate the isolation-by-distance relationship between the estimates of F_ST_/ (1 − F_ST_) and the natural logarithm of geographic distance. Geographical distance (in km) was measured using Google Earth (http://earth.google.com) based on a straight line connecting each pair of sampled populations.

STRUCTURE 2.3.3 [33] was used to investigate differentiation across the four Chinese egret populations. The analyses were performed using an admixture model with correlated allele frequencies, and testing numbers of clusters (K) ranged from 1 to 4 with 20 runs per *K* and a burn-in of 200,000 and 1,200,000 reps after the burn-in. The results were then uploaded to the Structure Harvester server (http://taylor0.biology.ucla.edu/structureHarvester/), which selects the number of clusters by simultaneously evaluating posterior probability and the Delta *K* statistic of [34]; the final of plots of population structure based on the most likely value of K were plotted graphically in CLUMPAK (http://clumpak.tau.ac.il/) [35].

## Results

### Polymorphism of TLRs

In this study, seven TLR (TLR1LB, TLR2A, TLR3, TLR4, TLR5, TLR7, and TLR15) sequences were characterized in the vulnerable Chinese egret and the common little egret (S1 Appendix). The NJ trees indicated that all TLR sequences in these two egrets were consistent with those in other species (house finch, lesser kestrel, New Zealand robin, white-winged flufftail, African penguin, little egret and Chinese egret) previously reported (S1 Fig). All of the LRRs regions of TLR1LA, TLR1LB, TLR3, TLR4, TLR5, TLR7, and TLR15 were successfully amplified in all 120 individuals of the Chinese egret and 20 individuals of the little egret. No stop codons or frameshift mutations were detected in these sequences of TLRs. All of the SNPs of TLR genes in the Chinese egret were diallelic. In the little egret, most of the SNPs were diallelic, except for one triallelic site that was detected in TLR2A

**Fig 2:**
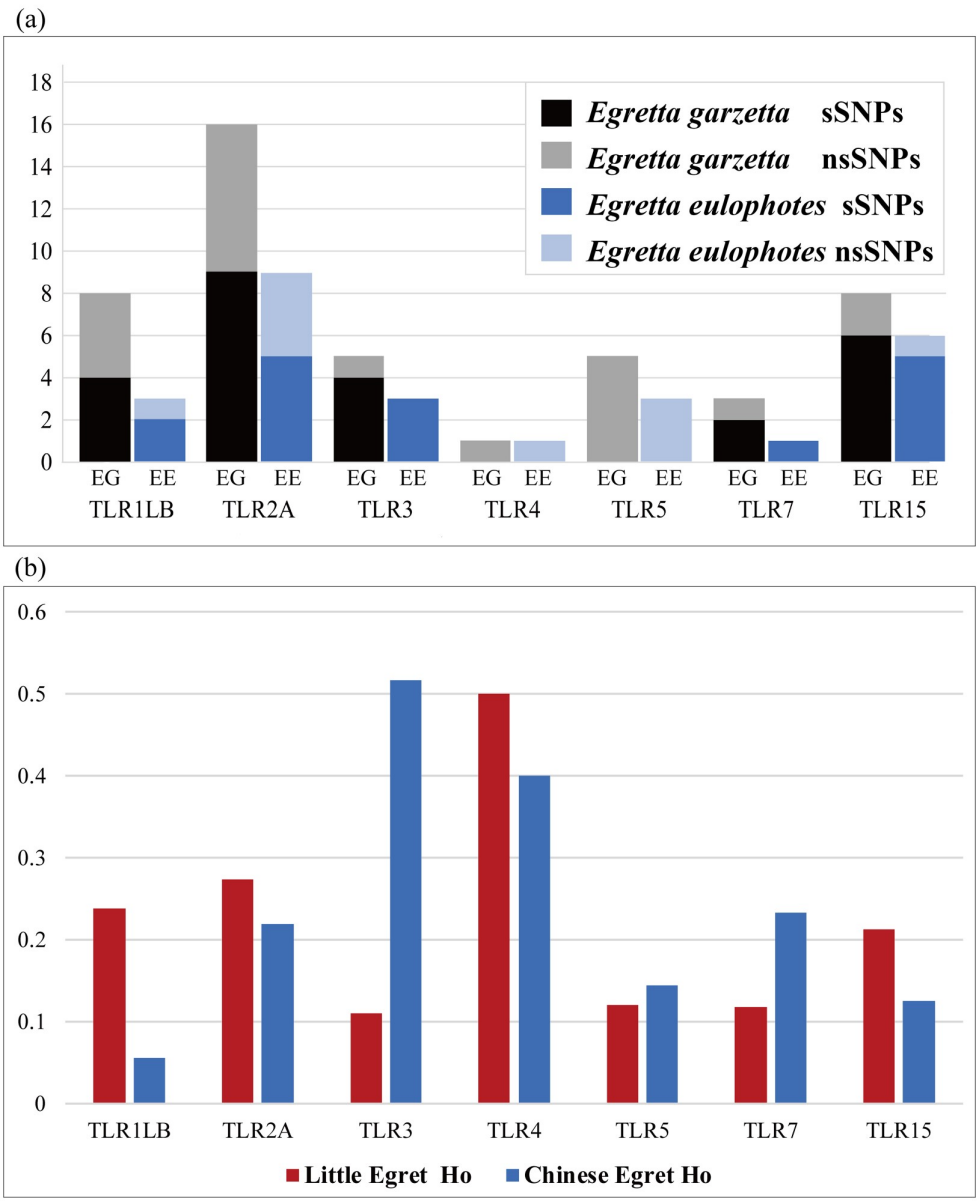
(a) The number of sSNPs and nsSNPs of seven TLRs (TLR1LB, TLR2A, TLR3, TLR4, TLR5, TLR7 and TLR15) in the Chinese egret (EE: *Egretta eulophotes*) and little egret (EG: *Egretta garzetta*). (b) TLR *Ho* (observed heterozygosity) comparison between the Chinese egret and little egret using the same individuals (N = 20).

In total, we found 26 (synonymous (s):non-synonymous (ns) = 16:10) and 45 (s:ns = 25:20) SNPs in the Chinese egret and little egret, respectively (Table 1), and the number of non-synonymous SNPs for each TLR in the Chinese egret was less than that of the little egret (Fig 2a). In the Chinese egret, TLR2A had the highest number of SNPs (n = 9), while the least numbers of SNPs occurred in TLR4 and TLR7 (n = 1). Concerning different populations, MTS and RY had the largest number of SNPs (n = 22), and the number of SNPs in the XCY population was the least (n = 17). All of the TLR genes contained at least one non-synonymous base substitution, except for TLR3. In comparison with the little egret, TLR2A also had the largest number of SNPs (n = 15) and the least number of SNPs in TLR4 (n = 1); all 45 SNPs comprised 25 synonymous and 20 non-synonymous substitutions, with 21 and 40 SNPs in the XY and MTS populations, respectively. In addition, TLR4 and TLR5 were the loci which showed higher number of nonsynonymous than synonymous SNPs (Table 1).

**Table 1:** Polymorphisms in Chinese egret Toll-like receptors.

Population	N	TLR1LB	TLR2A	TLR3	TLR4	TLR5	TLR7	TLR15	Total
Little egret (XY)	10	2(3)	7(2)	0(0)	0(1)	0(1)	0(1)	4(0)	**13(8)**
Little egret (MTS)	10	4(1)	7(6)	4(1)	0(1)	0(4)	2(1)	6(2)	**23(17)**
**Total**	**20**	**4(4)**	**9(6)**	**4 (1)**	**0(1)**	**0(5)**	**2(1)**	**6(2)**	**25(20)**
Chinese egret (XCY)	30	0(0)	3(4)	3(0)	0(1)	0(2)	1(0)	3(0)	**10(7)**
Chinese egret (RY)	30	1(1)	5(4)	3(0)	0(1)	0(3)	1(0)	4(1)	**14(8)**
Chinese egret (MTS)	30	2(1)	3(4)	3(0)	0(1)	0(2)	1(0)	5(0)	**14(8)**
Chinese egret (XRT)	30	2(0)	2(4)	3(0)	0(1)	0(2)	1(0)	4(0)	**12(7)**
**Total**	**120**	**2(1)**	**5(4)**	**3(0)**	**0(1)**	**0(3)**	**1(0)**	**5(1)**	**16(10)**

Synonymous SNPs are indicated outside of parentheses, and non-synonymous SNPs in the coding regions are indicated in parentheses.

### Tests for selection

All TLR genes showed more synonymous than non-synonymous mutations except for TLR5 (dn/ds = 48.60) (dn/ds of TLR4 and TLR7 could not be estimated by DataMonkey due to less than three unique sequences) (Table 2). Based on any two independent models (from SLAC, FUBAR and MEME), no positively selected sites were detected in any of TLRs. The negative/purifying selection sites were identified in most of the TLRs except for TLR5 (based on SLAC and FUBAR). Based on the FUBAR model, three positively selected sites were detected in TLR2A and TLR5. Tajima’s D test was not significant for any locus except for TLR3 (2.933, *p*<0.01), indicating that TLR3 evolved under balancing selection.

**Table 2:** Selection (characterized by non-synonymous (d_N_) and synonymous (d_S_) substitution rates) and polymorphism estimates (Watterson’s estimator of the population mutation rate (θw), the average number of nucleotide differences between alleles (k) and Tajima’s D test) for Chinese egret Toll-like receptors (TLR1LB, TLR2A, TLR3, TLR4, TLR5, TLR7, and TLR15).

Locus	Fragment length(aa)	*d*_S_ (*d*_N_)	*d*_*N*_*/d*_*S*_	Positive selection			Negative selection		Polymorphic estimates		
				SLAC	FUBAR	MEME	SLAC	FUBAR	K	*θ*_*w*_	Tajima’s D
TLR1LB	220	**2(1)**	0.217	0	0	0	0	1 (132)	0.283	0.0009	−0.859^ns^
TLR2A	294	**5(4)**	0.306	0	^*a*^2 (224, 284)	0	2 (53,281)	5 (53, 104, 171, 172, 281)	2.820	0.0019	1.652^ns^
TLR3	256	**3(0)**	0	0	0	0	1 (246)	3 (2, 235, 246)	1.504	0.0007	2.933 ^**< 0.01**^
TLR4	257	**0(1)**	^+^N/A	^+^N/A	^+^N/A	^+^N/A	^+^N/A	^+^N/A	0.464	0.0002	1.644^ns^
TLR5	315	**0(3)**	48.60	0	1 (280)	0	0	0	0.445	0.0006	−0.357^ns^
TLR7	307	**1(0)**	^+^N/A	^+^N/A	^+^N/A	^+^N/A	^+^N/A	^+^N/A	0.208	0.0002	0.126^ns^
TLR15	247	**5(1)**	0.114	0	0	0	1 (224)	2 (101, 224)	0.924	0.0015	−0.381^ns^

*d*_*N*_*/d*_*S*_ was calculated using the SLAC model implemented in the DataMonkey Web Server; ^+^N/A indicates that selection tests are not available because of less than three unique sequences. *a* the number of positively/negatively selected sites is indicated outside of parentheses, and the numbers inside of parentheses indicates the sites’ exact position on the amino acid fragment. The superscript in the Tajima’s D column is the *P*-value (statistical significance of *P* < 0.05).

### Comparison of TLR genetic diversity

**Fig 3:**
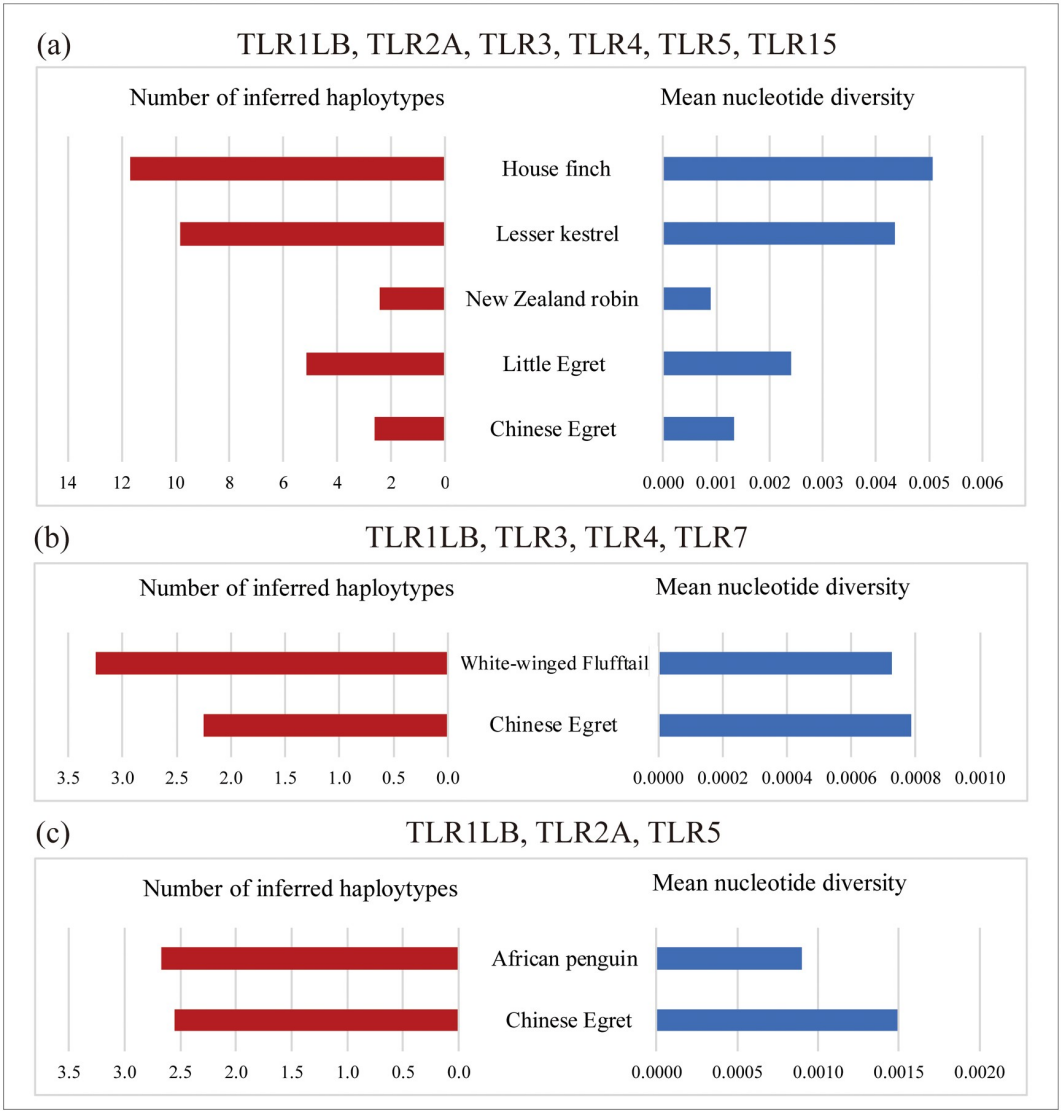
(a) Variance in estimates of *h* and mean nucleotide diversity *π* among six avian species calculated from six TLRs (TLR1LB, TLR3, and TLR4). (b) Variance in estimates of *h* and π between two avian species calculated from four TLRs (TLR1LB, TLR3, TLR4, and TLR7). (c) Variance in estimates of *h* and π between two avian species calculated from three TLRs (TLR1LB, TLR2A, and TLR5). *h* is the number of inferred haplotypes (indicated on the right in blue bars); *π* is the mean nucleotide diversity (indicated on the left in red bars).

For the Chinese egret, detailed diversity statistics of seven TLR genes within the four populations are summarized in Table 3. TLR diversity of four populations was found to be similar. The RY population had the highest level of genetic diversity (Ho = 0.285; He = 0.270; uHe = 0.274), and the MTS population had the lowest genetic diversity (Ho = 0.191; He = 0.243; uHe = 0.231). The XY population of the little egret had the lowest TLR diversity (Ho = 0.207; He = 0.156; uHe = 0.164) within the two little egret populations (n = 20) and four Chinese egret populations (n = 120). To accurately compare the differences in TLR genetic diversity between the Chinese egret and little egret, 20 samples of the Chinese egret were randomly sampled from 120 samples three times, and the average value was obtained. The results showed that for most TLR genes, Ho of the Chinese egret was lower than that of the little egret, except for TLR3, TLR5, and TLR7 (S2 Table, Fig 2b). We also conducted a comparison between the TLRs of the Chinese egret and other avian species, including three threatened species (the white-winged flufftail (*Sarothrura ayresi*), the African penguin (*Spheniscus demersus*), and New Zealand robin (*Petroica australis rakiura*) and three more common and widespread species (the little egret (*Egretta garzetta*), the house finch (*Carpodacus mexicanus*) and the lesser kestrel (*Falco naumanni*). Based on seven TLRs (TLR1LB, TLR2A, TLR3, TLR4, TLR5, TLR7, and TLR15) in these five species, we conducted a comparison of mean nucleotide diversity (π) and number of inferred haplotypes (*h*) (Table 4, Fig 3). The number of haplotypes (*h*) ranged from 2 to 20 with a mean *h* = 6.2, and nucleotide diversity ranged from 0 to 0.0100, with a mean π = 0.0028. Both *h* and π for the Chinese egret are close to the values for the New Zealand robin but were lower than in the more common species, the little egret, the house Finch and the lesser kestrel (Fig 3a). We also performed a comparison for the Chinese egret, the white-winged flufftail and the African penguin based on different TLR groups; the number of haplotypes (*h*) in the Chinese egret was lower than in the white-winged flufftail and African penguin, although mean nucleotide diversity (π) was higher than in either species (Fig 3b and 3c).

**Table 3:** Genetic diversity statistics of the TLRs in four Chinese egret populations and two little egret populations.

Locus		Population	N	*h*	*G*d	π	*H*o	*H*e	*uHe*
**TLR1LB**	**Chinese egret**	XCY	30	1	0	0	0.022	0.022	0.022
		RY	30	3	0.131	0.0003	0.133	0.123	0.125
		MTS	30	3	0.246	0.0008	0.089	0.084	0.085
		XRT	30	3	0.246	0.0006	0.133	0.120	0.122
		**Total**	**120**	**4**	**0.158**	**0.0004**	**0.094**	**0.087**	**0.089**
	**little egret**	MTS	10	6	0.889	0.0031	0.175	0.199	0.209
		XY	10	6	0.867	0.0028	0.300	0.203	0.213
		**Total**	**20**	**11**	**0.937**	**0.0034**	**0.238**	**0.201**	**0.211**
**TLR2A**	**Chinese egret**	XCY	30	6	0.669	0.0032	0.289	0.328	0.334
		RY	30	10	0.777	0.0035	0.330	0.348	0.354
		MTS	30	6	0.607	0.0031	0.159	0.293	0.298
		XRT	30	7	0.683	0.0032	0.263	0.257	0.262
		**Total**	**120**	**15**	**0.690**	**0.0032**	**0.260**	**0.307**	**0.312**
	**little egret**	MTS	10	8	0.956	0.0052	0.293	0.279	0.294
		XY	10	6	0.867	0.0037	0.253	0.195	0.205
		**Total**	**20**	**12**	**0.916**	**0.0045**	**0.273**	**0.237**	**0.249**
**TLR3**	**Chinese egret**	XCY	30	5	0.630	0.0020	0.489	0.451	0.459
		RY	30	6	0.605	0.0019	0.533	0.473	0.481
		MTS	30	6	0.667	0.002	0.489	0.496	0.504
		XRT	30	6	0.747	0.0019	0.511	0.497	0.505
		**Total**	**120**	**7**	**0.664**	**0.0020**	**0.506**	**0.479**	**0.487**
	**little egret**	MTS	10	6	0.889	0.0022	0.220	0.233	0.245
		XY	10	0	0	0	0	0	0
		**Total**	**20**	**6**	**0.579**	**0.0012**	**0.110**	**0.117**	**0.123**
**TLR4**	**Chinese egret**	XCY	30	2	0.508	0.0007	0.567	0.433	0.440
		RY	30	2	0.408	0.0006	0.467	0.444	0.452
		MTS	30	2	0.370	0.0005	0.367	0.299	0.305
		XRT	30	2	0.497	0.0006	0.500	0.433	0.440
		**Total**	**120**	**2**	**0.464**	**0.0006**	**0.475**	**0.402**	**0.409**
	**little egret**	MTS	10	2	0.467	0.0006	0.600	0.480	0.505
		XY	10	2	0.533	0.0007	0.400	0.320	0.337
		**Total**	**20**	**2**	**0.521**	**0.0007**	**0.500**	**0.400**	**0.421**
**TLR5**	**Chinese egret**	XCY	30	3	0.246	0.0005	0.178	0.154	0.157
		RY	30	5	0.411	0.0006	0.233	0.196	0.199
		MTS	30	4	0.407	0.0006	0.200	0.200	0.203
		XRT	30	3	0.131	0.0002	0.044	0.043	0.044
		**Total**	**120**	**5**	**0.299**	**0.0005**	**0.164**	**0.148**	**0.151**
	**little egret**	MTS	10	4	0.733	0.0013	0.140	0.183	0.193
		XY	10	2	0.467	0.0005	0.100	0.089	0.094
		**Total**	**20**	**5**	**0.626**	**0.0010**	**0.120**	**0.136**	**0.143**
**TLR7**	**Chinese egret**	XCY	30	2	0.186	0.0002	0.100	0.095	0.097
		RY	30	2	0.186	0.0002	0.300	0.255	0.259
		MTS	30	2	0.186	0.0002	0.200	0.180	0.183
		XRT	30	2	0.287	0.0003	0.233	0.299	0.305
		**Total**	**120**	**2**	**0.208**	**0.0002**	**0.208**	**0.207**	**0.211**
	**little egret**	MTS	10	4	0.644	0.0008	0.133	0.123	0.13
		XY	10	2	0.467	0.0005	0.100	0.125	0.132
		**Total**	**20**	**4**	**0.553**	**0.0007**	**0.117**	**0.124**	**0.131**
**TLR15**	**Chinese egret**	XCY	30	6	0.543	0.0010	0.150	0.148	0.151
		RY	30	7	0.547	0.0013	0.161	0.133	0.136
		MTS	30	6	0.579	0.0016	0.106	0.143	0.145
		XRT	30	4	0.434	0.0012	0.161	0.147	0.149
		**Total**	**120**	**9**	**0.520**	**0.0013**	**0.144**	**0.143**	**0.145**
	**little egret**	MTS	10	7	0.911	0.0039	0.188	0.256	0.269
		XY	10	4	0.711	0.0017	0.238	0.166	0.174
		**Total**	**20**	**10**	**0.905**	**0.0031**	**0.213**	**0.211**	**0.222**

*N* Total number of haplotypes, *h* the number of haplotypes, *Gd* gene diversity, *π* nucleotide diversity, *Ho* observed heterozygosity, *He* expected heterozygosity, *uHe* unbiased expected heterozygosity.

**Table 4:** Comparison of TLR polymorphisms between Chinese egret and other avian species.

Species	Genes	*N*	SNPs	*h*	π	Reference
**Chinese egret** (*Egretta eulophotes*) ^a^	TLR1LB	10	1	2	0.0004	This study
	TLR2A	10	7	4	0.0033	
	TLR3	10	3	3	0.0017	
	TLR4	10	1	2	0.0007	
	TLR5	10	2	2	0.0009	
	TLR7	10	1	2	0.0004	
	TLR15	10	3	3	0.0011	
**Little egret** (*Egretta garzetta*) ^b^	TLR1LB	10	6	7	0.0034	This study
	TLR2A	10	10	6	0.0044	
	TLR3	10	5	4	0.0017	
	TLR4	10	1	2	0.0007	
	TLR5	10	3	4	0.0006	
	TLR7	10	2	3	0.0006	
	TLR15	10	7	8	0.0036	
**White-winged flufftail** (*Sarothrura ayresi*)	TLR1LB	10	5	5	0.0014	Dalton et al., 2016
	TLR2A	-	-	-	-	
	TLR3	10	1	2	0.0004	
	TLR4	10	0	1	0.0000	
	TLR5	-	-	-	-	
	TLR7	10	4	5	0.0011	
	TLR15	-	-	-	-	
**African penguin** (*Spheniscus demersus*)	TLR1LB	10	2	3	0.0005	Dalton et al., 2016
	TLR2A	10	4	3	0.0020	
	TLR3	-	-	-	-	
	TLR4	-	-	-	-	
	TLR5	10	1	2	0.0002	
	TLR7	-	-	-	-	
	TLR15	-	-	-	-	
**New Zealand robin** (*Petroica australis rakiura*)	TLR1LB	10	3	2	0.0016	Grueber et al., 2012
	TLR2A	10	1	2	0.0005	
	TLR3	9	0	1	0.0000	
	TLR4	10	4	5	0.0027	
	TLR5	10	2	3	0.0005	
	TLR7	10	3	≥ 2	N/A	
	TLR15	10	1	2	0.0000	
**Lesser kestrel** (*Falco naumanni*)	TLR1LB	8	16	15	0.0039	Alcaide and Edwards, 2011
	TLR2A	8	6	5	0.0040	
	TLR3	8	1	2	0.0009	
	TLR4	8	6	7	0.0030	
	TLR5	8	20	16	0.0043	
	TLR7	8	3	4	0.0017	
	TLR15	8	43	14	0.0100	
**House finch** (*Carpodacus mexicanus*)	TLR1LB	8	25	20	0.0067	Alcaide and Edwards, 2011
	TLR2A	8	13	8	0.0067	
	TLR3	8	11	9	0.0038	
	TLR4	8	16	14	0.0049	
	TLR5	8	2	3	0.0001	
	TLR7	8	27	15	0.0077	
	TLR15	8	35	16	0.0082	

^*a*^ 10 samples were randomly sampled from 120 samples three times, and the average value was obtained.

^*b*^ 10 samples were randomly sampled from 20 samples three times, and the average value was obtained.

*N* Number of samples, *h* the number of haplotypes, *π* mean nucleotide diversity.

### Population differentiation and population structure

**Fig 4:**
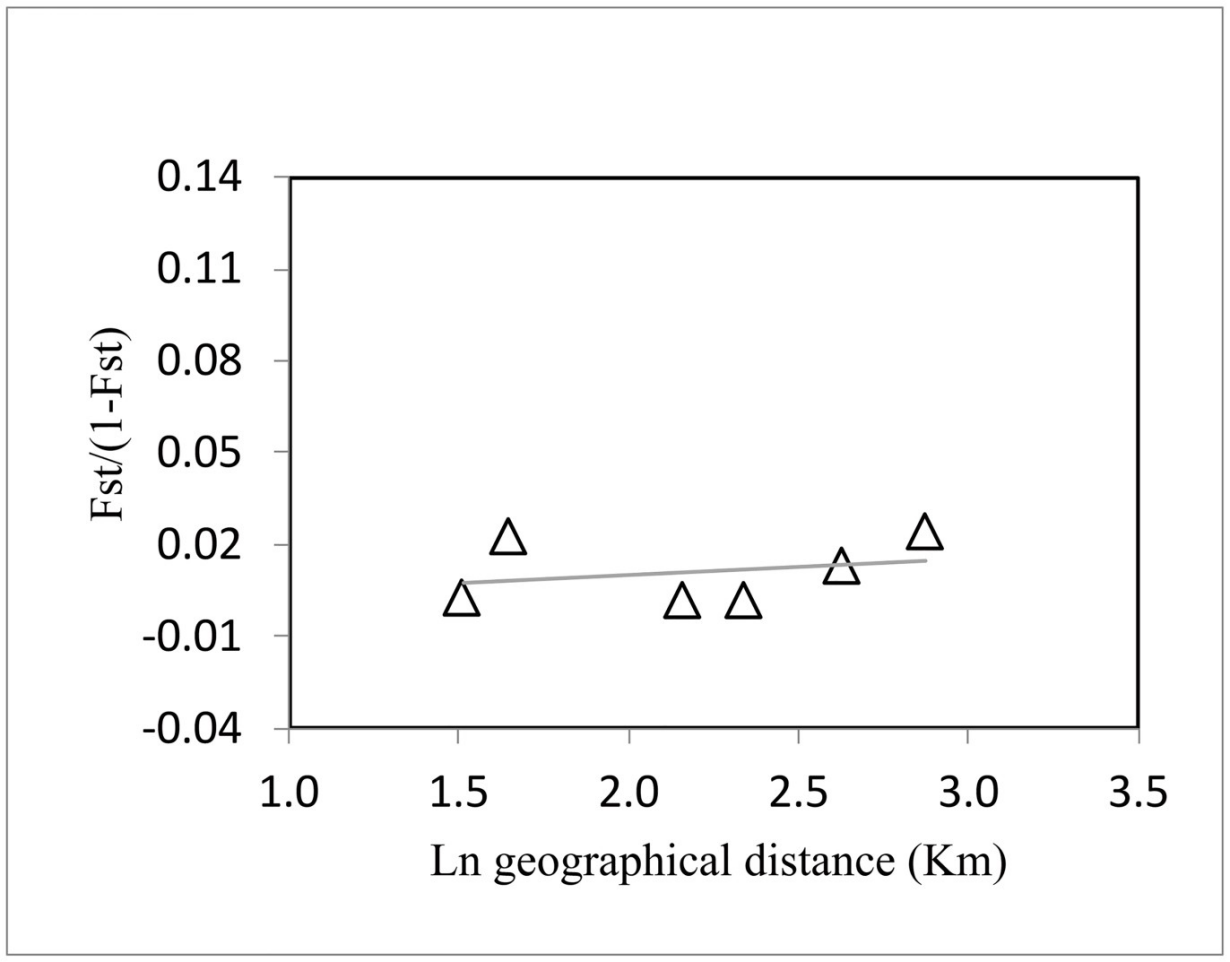
Isolation-by-distance, with pairwise comparisons of the four Chinese egret populations. Filled triangles represent pairwise comparison values based on estimated molecular distances.

**Fig 5:**
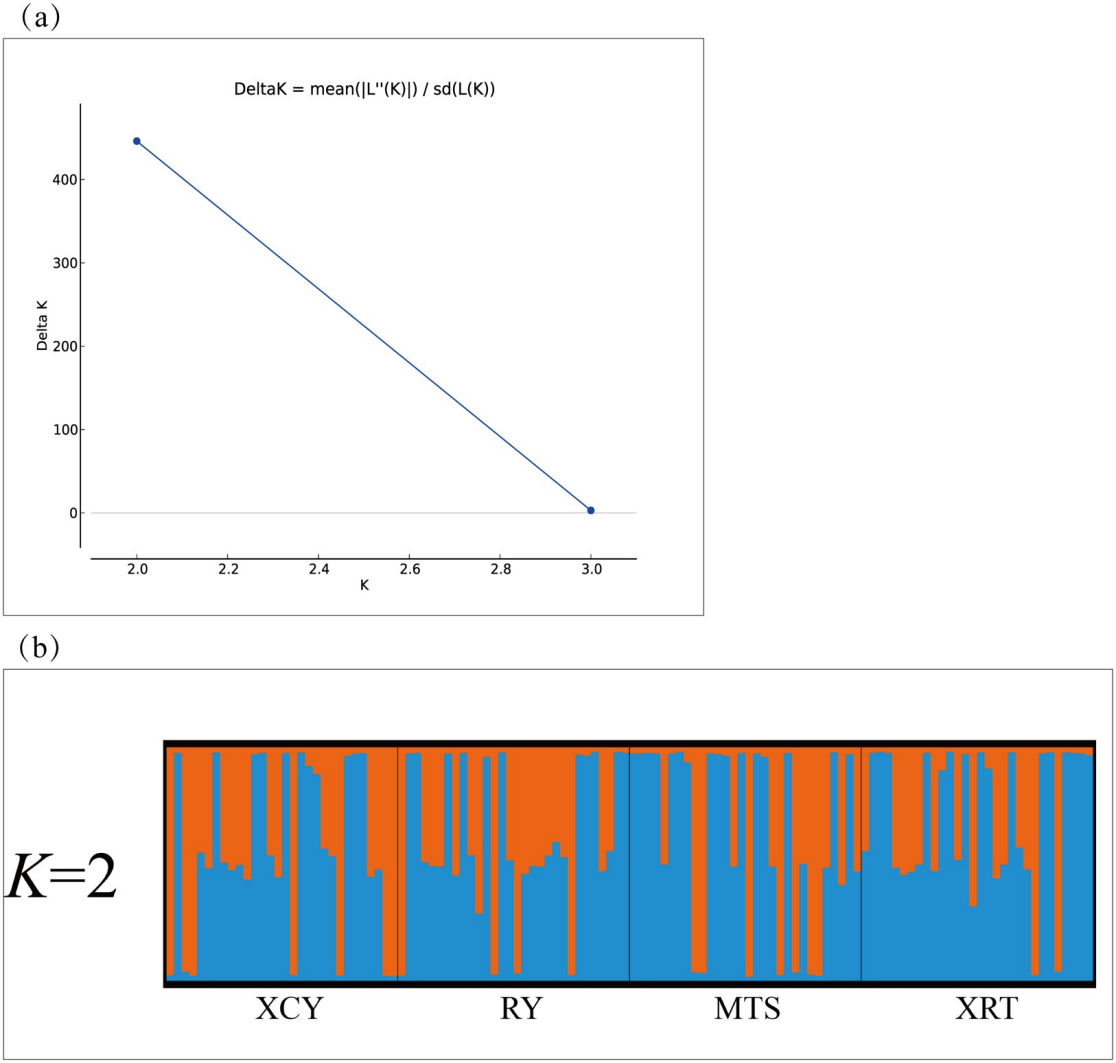
Results from structure analyses using 26 SNPs of TLRs (TLR1LB, TLR2A, TLR3, TLR4, TLR5, TLR7, and TLR15) in four Chinese egret populations. Use of the Evanno et al. (2005) ΔK approach (a) suggested that there were two clusters. However, assignment probabilities of individuals to these clusters were nearly uniform (b).

The AMOVA revealed low but significant genetic differentiation (F_ST_ = 0.011, *p* = 0.043 < 0.05), and a high proportion of the total genetic variance was attributable to variations within populations (98.87%). Considering TLR3 and TLR5, low but significant genetic differentiation was present among the four populations at the 0.05 level (F_ST_ = 0.032, 0.023, respectively; all *p* < 0.05) (Table 5). To further assess the genetic differentiation between populations, pairwise comparisons of F_ST_ values were calculated (Table 6). For all of the TLR loci, pairwise F_ST_ values based on estimated molecular distances ranged from 0.002 ‒ 0.024, and no significant genetic differentiation was indicated (*p* < α, α = 0.05/6 after Bonferroni-adjusted) among these populations (Table 6). The Mantel test indicated that there was no significant isolation-by-distance relationship at the TLR loci, comparing the F_ST_/(1 − F_ST_) value based on estimated molecular distances with the natural logarithm of the geographic distance (*r* = 0.283, *p* = 0.300) (Fig 4). STRUCTURE analyses provided no evidence of separate genetic structure (Fig 5b); among the values of K investigated, the greatest average likelihood score was observed for K = 2, and the use of the Δ*K* approach also suggested that there were two clusters (Fig 5a).

**Table 5:** Analysis of molecular variance (AMOVA) of the TLRs (TLR1LB, TLR2A, TLR3, TLR4, TLR5, TLR7, and TLR15) in the Chinese egret.

Locus	Source	df	Variance (%)	Percentage variation	*Fst*	*P*
TLR1LB	Among Pops	3	0.001	1.07%	0.011	0.143
	Within Pops	236	0.133	98.93%		
TLR2A	Among Pops	3	0.007	0.53%	0.005	0.242
	Within Pops	236	1.404	99.47%		
TLR3	Among Pops	3	0.024	3.20%	0.032	0.023*
	Within Pops	236	0.731	96.80%		
TLR4	Among Pops	3	0.001	0.72%	0.007	0.236
	Within Pops	236	0.205	99.28%		
TLR5	Among Pops	3	0.005	2.30%	0.023	0.043*
	Within Pops	236	0.226	97.70%		
TLR7	Among Pops	3	0.002	1.54%	0.015	0.131
	Within Pops	236	0.105	98.46%		
TLR15	Among Pops	3	0.000	0%	-0.010	0.925
	Within Pops	236	0.436	100%		
**TLRs**	Among Pops	3	0.037	1.13%	0.011	0.043*
	Within Pops	236	3.240	98.87%		

* Statistical significance of *P* < 0.05.

**Table 6:** Pairwise F_ST_ values (below diagonal) and significance (above diagonal) for each location of the sampled Chinese egret populations.

Locus	Population	XCY	RY	MTS	XRT
TLR1LB	XCY		0.039	0.176	0.028
	RY	0.030		0.430	0.336
	MTS	0.014	−0.010		0.296
	XRT	0.059	−0.003	−0.004	
TLR2A	XCY		0.320	0.308	0.055
	RY	−0.008		0.335	0.140
	MTS	0.001	−0.007		0.318
	XRT	0.035	0.015	−0.005	
TLR3	XCY		0.002	0.214	0.113
	RY	0.120		0.083	0.133
	MTS	0.009	0.032		0.315
	XRT	0.020	0.020	−0.015	
TLR4	XCY		0.249	0.135	0.321
	RY	−0.016		0.093	0.246
	MTS	0.030	0.041		0.133
	XRT	−0.017	−0.016	0.030	
TLR5	XCY		0.374	0.376	0.052
	RY	−0.012		0.286	0.006
	MTS	−0.007	−0.016		**P=0.004<α**
	XRT	0.048	0.075	0.095	
TLR7	XCY		0.130	0.483	0.046
	RY	0.038		0.273	0.218
	MTS	0.001	−0.005		0.290
	XRT	0.067	−0.013	0.012	
TLR15	XCY		0.384	0.384	0.358
	RY	−0.013		0.343	0.369
	MTS	−0.011	−0.015		0.343
	XRT	−0.012	−0.005	−0.005	
**TLRs**	XCY		0.031	0.298	0.030
	RY	0.023		0.281	0.092
	MTS	0.002	0.003		0.311
	XRT	0.024	0.013	0.002	

α = 0.05/6 after Bonferroni-adjusted.

## Discussion

### SNPs

In this study, the Chinese egret had a low level of SNPs in the TLRs compared to the little egret, as in previous studies with other endangered or critically endangered species [7, 21, 36]. Apart from the conservativeness of TLR-ligand binding and low frequency of naturally occurring variation (protein-encoding genes) in TLRs for some taxa [37], the low level of TLR population polymorphism may be caused by inbreeding, as the Chinese egret declined sharply before the 19th century. Therefore, the low TLR polymorphism observed in this vulnerable species is most likely to be the result of negative/purifying selection acting on the TLRs and inbreeding depression in small populations of the vulnerable species compared with the larger population sizes of the widely distributed species [38]. Since the polymorphisms at TLR loci have a direct impact on resistance/susceptibility to pathogen infection across a range of vertebrate groups, low TLR polymorphism may be associated with a higher susceptibility to infectious disease [7, 22, 39–44]. For example, in humans, special SNPs at TLR1 and TLR2 contribute to the course of sepsis [45, 46]; SNP variants of TLR3, TLR4, and TLR7 are promising biomarkers of liver cirrhosis and cancer associated with HBV and HCV infection [47, 48]; heterozygous variants for single nucleotide polymorphisms (SNPs) in TLR5 were associated with higher levels of Interferon-gamma secretion [49]. In cattle and sheep, the various polymorphisms in TLR2 are associated with reduced SCC (Somatic Cell Count) or increased residence to mastitis [50, 51]. In chickens, TLR15 gene polymorphism is involved in resistance to *Salmonella enterica* in Chinese native chicken breeds [52].

### Selection of TLRs

Our analysis revealed purifying selection acting on TLRs in the Chinese egret except for TLR5. No positively selected sites were detected by either method. Also, no episodic diversifying selected sites were detected under MEME, although episodic positive selection played an important role in the evolution of most avian TLRs [53]. Moreover, at least one negative site (n = 1‒5) was detected in most TLRs (TLR1LB, TLR2A, TLR3, and TLR15) except for TLR5 (n = 0) based on both SLAC and FUBAR models, indicating that purifying selection was more likely to be acting on those loci. In the TLRs of the Chinese egret, TLR5 was the unique gene acted on by strong positive selection, as a significant excess of non-synonymous alterations over synonymous substitutions was observed, and only one positively selected site was detected (based on FUBAR), consistent with the findings of Velová et al. [15].

### Genetic diversity

Four in seven of TLR genetic diversity in the Chinese egret was lower compared to the common little egret in China, except for TLR3, TLR5, and TLR7 (Table 3). This result was similar to the critically endangered white-winged flufftail (*Sarothrura ayresi*), in which genetic diversity was lower than in the common red-chested flufftail (*Sarothrura rufai*) in South Africa [7]. Because population size is the key factor in genetic diversity assessments, we randomly sampled 20 samples from 120 individuals three times to accurately compare the Chinese egret sample with the little egret (n = 20) (S2 Table, Fig 2b). The result is consistent with the above interpretation. In addition, the Chinese egret’s level of genetic diversity was lower compared to other common species such as the house finch and lesser kestrel, and close to the New Zealand robin. Compared with other endangered species, the number of haplotypes in the Chinese egret was still lower than in the white-winged flufftail and African penguin, but the mean nucleotide diversity was higher than in either species. A majority (57 percent) of TLR genetic diversity in the Chinese egret were low, which may be due to their endangered mechanism resulted from interior factors integrated with exterior factors such as low gene flow, inbreeding depression, small populations, habitat contraction or fragmentation, diversity of co-evolving pathogens, and many other factors [10, 13, 21, 54–58].

The heterozygosity of TLR3 (Ho = 0.517) in a migratory bird, the Chinese egret, is apparently higher than the heterozygosity of TLR7 (Ho = 0.233), suggesting different pathogen selection pressures between the TLR3 and TLR7 genes [59]. In the innate immunity mechanisms of TLRs, compared to bacterial-sensing TLRs such as TLR4 and TLR5, the viral-sensing TLR3 and TLR7 detect structurally invariant RNA molecules regardless of their precise sequence (TLR3 detects dsRNA, and TLR7 binds ssRNA) [44, 60]. Long-distance migratory wading birds might suffer more immunological suppression and infection risk from pathogens than residents and non-migratory birds, as they experience different environments (breeding region, stopover sites, and wintering ground) and different pathogen communities during aggregating migration [61]. Under such circumstances, there may be a negative impact on the lives and survival of migratory waterfowl, especially endangered species [62–64], leading to strong immunologic mechanisms that guard against invading viruses such as avian influenza virus [43, 65, 66].

The heterozygosity of both TLR3 (Ho = 0.517) and TLR7 (Ho = 0.233) in the Chinese egret were higher than in the little egret (TLR3 Ho = 0.110 and TLR7 Ho = 0.117) (S2 Table). In contrast, the Chinese egret’s TLR3 (s:ns = 3:0) and TLR7 (s:ns = 1:0) have lower SNPs than the same loci in the little egret (TLR3 s:ns = 4:1 and TLR7 s:ns = 2:1) (Table 1). Considering these results, we hypothesized that the Chinese egret population can still ensure its ability to resist viruses through heterozygote advantage; i.e., a mechanism of balancing selection acts on immunogenetic variation in various species, thereby ultimately improving individual survival and reproduction [22, 67–70]. In the little egret, the heterozygosity and SNPs of TLR3 (Ho = 0.220, s:ns = 4:1) and TLR7 (Ho = 0.133, s:ns = 2:1) in the MTS island population were higher than in the XY mainland population (TLR3 Ho = 0, s:ns = 0 and TLR7 Ho = 0.100, s:ns = 0:1) (Tables 1 and 3). The reason for the above is probably that the MTS island has many migratory avian species breeding together, which may lead the little egret population on MTS island to be exposed to more types of pathogens brought by other avian species, consequently increasing infection risk and adaptively generating higher genetic diversity [71, 72].

### Population differentiation and population structure

There was low but significant divergence in TLRs among populations, indicating the presence of a comparable level of gene flow among these four Chinese egret populations. This genetic exchange is most likely made by the individual movements among populations meeting in the same breeding, stopover or wintering sites during long-distance migration. AMOVA analysis showed low but significant F_ST_ values (F_ST_ = 0.011, *p* = 0.043 < 0.05) for overall TLR loci using molecular distances, and also low but significant F_ST_ values (*p* = 0.020 and 0.037) for TLR3 and TLR5 although, this was not supported by TLR1LB, TLR2A, TLR4, TLR7, or TLR15. Pairwise population analysis indicated that there was no significant genetic differentiation among the four populations, in accord with our previous study [5]. The patterns of isolation by distance (IBD) were also not detected in the present study, suggesting that the differentiation among XRT and MTS at TLR5 loci was not related to geographic distance. Moreover, Bayesian analysis in STRUCTURE showed that the four populations of this egret had high levels of admixture, consistent with our previous report that these four populations in China might be considered as a single adaptive unit for conservation [3].

In conclusion, this study reported the TLR genetic diversity in an Ardeid species by assessing seven TLR loci. Our analyses confirmed the hypothesis that both SNPs and the majority of genetic diversity of toll-like receptor genes in the vulnerable species, the Chinese egret, were lower than those in the more common and widely distributed species, the little egret, in accordance with previous reports on other threatened avian species. The selection test indicated TLRs, except for TLR5, were under purifying selection in TLR evolution, suggesting that low TLR genetic diversity in the Chinese egret may be caused by purifying selection. Moreover, among the four populations in this egret, molecular variance analysis showed low but significant genetic differentiation at TLR loci, pairwise population analysis indicated that there was no significant genetic differentiation, and Bayesian analysis revealed the populations had high levels of admixture in structure. Taking account of these three results together as a whole, the four populations of the Chinese egret in China should be considered as a single adaptive unit for conservation. These new results may provide fundamental TLR information for further studies on the conservation genetics of the Chinese egret and other Ardeids.

## Supporting information

S1 Fig Neighbor-joining trees showing the phylogenetic relationships of seven TLRs (TLR1LB, TLR2A, TLR3, TLR4, TLR5, TLR7, and TLR15) among seven avian species. The bootstrap values are displayed at each branch point.
(TIF)

S1 Table PCR primers for seven TLR genes in the Chinese egret and little egret. (DOCX)

S2 Table Observed and expected heterozygosity and unbiased heterozygosity estimates for every Toll-like receptor loci genotyped in the Chinese egret (*Egretta eulophotes*) and little egret (*Egretta garzetta*). *20 samples were randomly sampled from 120 samples three times, and the average value was obtained. *N* Number of samples, *Ho* mean observed heterozygosity, *He* mean expected heterozygosity, *uHe* unbiased expected heterozygosity.
(DOCX)

S1 Appendix Nucleotide sequence alignments of TLR genes in the Chinese egret (*Egretta eulophotes*) and little egret (*Egretta garzetta*). (XLSX)
