# Supplementary figures and images for: Genetic diversity of toll-like receptor genes in the vulnerable Chinese egret (Egretta eulophotes)
Source: PLoS One. 2020 May 29;15(5):e0233714. doi: 10.1371/journal.pone.0233714 (PMC7259618; doi:10.1371/journal.pone.0233714)

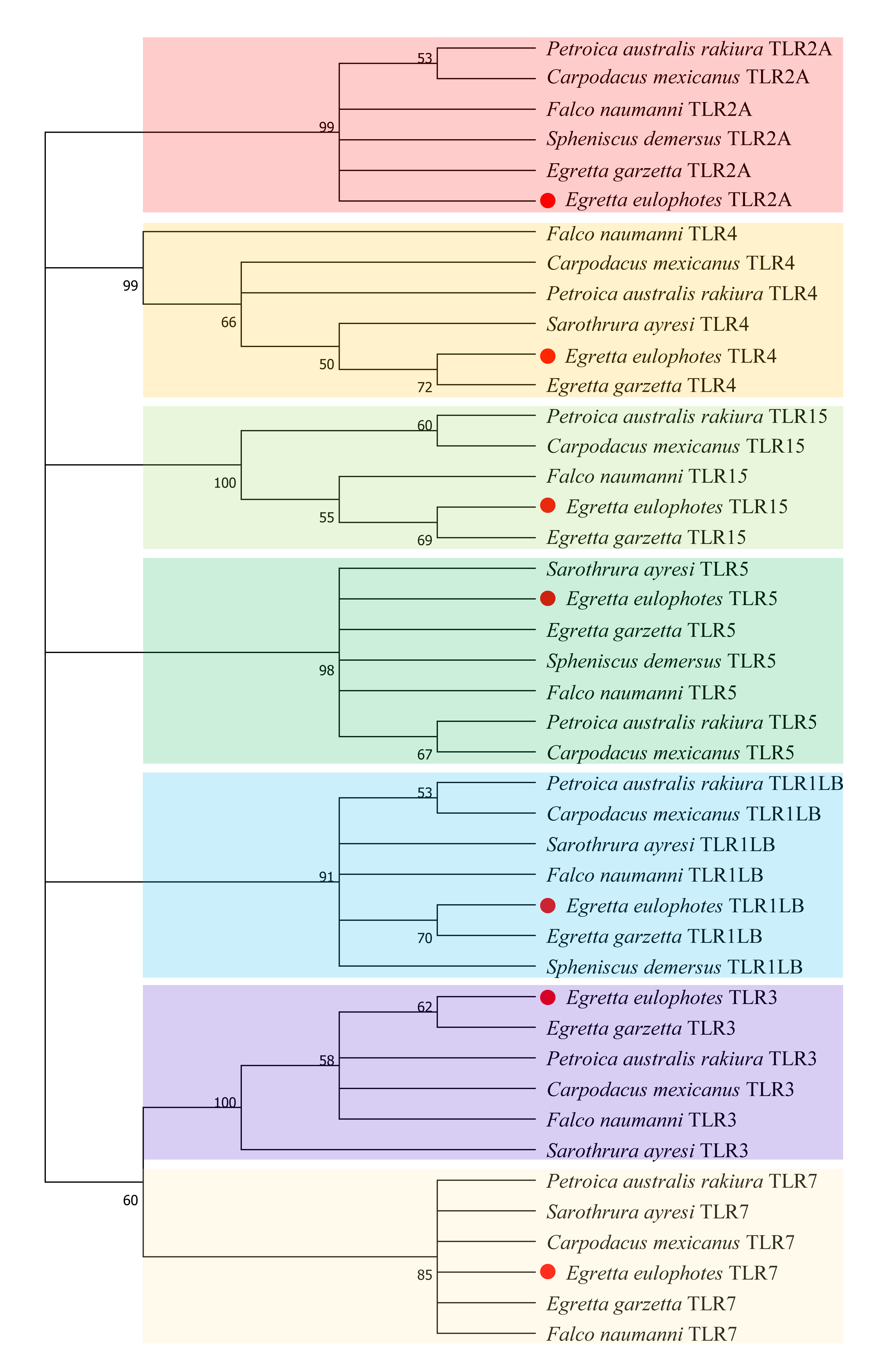

Supplement: S1 Fig — The bootstrap values are displayed at each branch point. (TIF) [file pone.0233714.s001.tif]
