# Supplementary material for: Genetic diversity of toll-like receptor genes in the vulnerable Chinese egret (Egretta eulophotes)
Source: PLoS One. 2020 May 29;15(5):e0233714. doi: 10.1371/journal.pone.0233714 (PMC7259618; doi:10.1371/journal.pone.0233714)
Supplement: S1 Table — (DOCX) [file pone.0233714.s002.docx]

**S1 Table. PCR primers for seven TLR genes in the Chinese egret and little egret**.

| Gene | Primers | Sequence | Size(bp) |
| --- | --- | --- | --- |
| TLR1LB | EgeuTLR1LBF | GAAAGCATTGACAGTGGAGAAA | 705 |
|  | EgeuTLR1LBR | TCTTGCAGTTCACAGGAACAC |  |
| TLR2A | EgeuTLR2AbF | GATTGATTGTGTGCTTGAGGGG | 927 |
|  | EgeuTLR2AbR | ATCAACGACACCACGAGGGA |  |
| TLR3 | EgeuTLR3F | CCAGGGACTGCAARGAACAA | 811 |
|  | EgeuTLR3R | ATGTTTCCAAAGTCGGGCCA |  |
| TLR4 | EgeuTLR4F | ACACCTCAATAGCTTCCGGC | 813 |
|  | EgeuTLR4R | CTGACTTGGCAGGAGGACAG |  |
| TLR5 | EgeuTLR5F | ATGGGTTCGGGATTTGGCTT | 988 |
|  | EgeuTLR5R | GCCACGCTAGTAGGCTCTTT |  |
| TLR7 | EgeuTLR7bF | CACAGTGCTTGACCTGTCCT | 980 |
|  | EgeuTLR7bR | GCAGAAAGCTGAGCGAGTTG |  |
| TLR15 | EgeuTLR15F | GAACTGTACGCATCAACGCC | 781 |
|  | EgeuTLR15R | TTTATCTGCAGGTGCGGGTT |  |
