# Supplementary material for: Genetic diversity of toll-like receptor genes in the vulnerable Chinese egret (Egretta eulophotes)
Source: PLoS One. 2020 May 29;15(5):e0233714. doi: 10.1371/journal.pone.0233714 (PMC7259618; doi:10.1371/journal.pone.0233714)
Supplement: S2 Table — *20 samples were randomly sampled from 120 samples three times, and the average value was obtained. N Number of samples, Ho mean observed heterozygosity, He mean expected heterozygosity, uHe unbiased expected heterozygosity. (DOCX) [file pone.0233714.s003.docx]

**S2 Table. Observed and expected heterozygosity and unbiased heterozygosity estimates for every Toll-like receptor loci genotyped in the Chinese egret (*Egretta eulophotes*) and little egret (*Egretta garzetta*).**

| **Species** | **N** | **Ho/He** | **TLR1LB** | **TLR2A** | **TLR3** | **TLR4** | **TLR5** | **TLR7** | **TLR15** |
| --- | --- | --- | --- | --- | --- | --- | --- | --- | --- |
| **Little egret** | 20 | Ho | 0.238 | 0.273 | 0.110 | 0.500 | 0.120 | 0.117 | 0.213 |
|  |  | He | 0.201 | 0.237 | 0.117 | 0.400 | 0.136 | 0.124 | 0.211 |
|  |  | uHe | 0.211 | 0.249 | 0.123 | 0.421 | 0.143 | 0.211 | 0.249 |
| **Chinese egret** | 20***** | Ho | 0.056 | 0.219 | 0.517 | 0.400 | 0.144 | 0.233 | 0.125 |
|  |  | He | 0.053 | 0.296 | 0.485 | 0.350 | 0.145 | 0.206 | 0.111 |
|  |  | uHe | 0.055 | 0.303 | 0.497 | 0.359 | 0.149 | 0.211 | 0.114 |

*****20 samples were randomly sampled from 120 samples for 3 times, and the average value was obtained.

*N* Number of samples, *Ho* mean observed heterozygosity, *He* mean expected heterozygosity, *uHe* unbiased expected heterozygosity.
